# Growth of Mouse Oocytes to Maturity from Premeiotic Germ Cells *In Vitro*

**DOI:** 10.1371/journal.pone.0041771

**Published:** 2012-07-24

**Authors:** Zhi-Peng Zhang, Gui-Jin Liang, Xi-Feng Zhang, Guo-Liang Zhang, Hu-He Chao, Lan Li, Xiao-Feng Sun, Ling-Jiang Min, Qing-Jie Pan, Qing-Hua Shi, Qing-Yuan Sun, Massimo De Felici, Wei Shen

**Affiliations:** 1 Key Laboratory of Animal Reproduction and Germplasm Enhancement in Universities of Shandong, Qingdao Agricultural University, Qingdao, China; 2 Hefei National Laboratory for Physical Sciences at Microscale and School of Life Sciences, University of Science and Technology of China, Hefei, Anhui, China; 3 State Key Laboratory of Reproductive Biology, Institute of Zoology, Chinese Academy of Sciences, Beijing, China; 4 Department of Biomedicine and Prevention, University of Rome ‘Tor Vergata’, Rome, Italy; Baylor College of Medicine, United States of America

## Abstract

In the present study, we established an *in vitro* culture system suitable for generating fertilizable oocytes from premeiotic mouse female germ cells. These results were achieved after first establishing an *in vitro* culture system allowing immature oocytes from 12–14 day- old mice to reach meiotic maturation through culture onto preantral granulosa cell (PAGC) monolayers in the presence of Activin A (ActA). To generate mature oocytes from premeiotic germ cells, pieces of ovaries from 12.5 *days post coitum* (dpc) embryos were cultured in medium supplemented with ActA for 28 days and the oocytes formed within the explants were isolated and cocultured onto PAGC monolayers in the presence of ActA for 6–7 days. The oocytes were then subjected to a final meiotic maturation assay to evaluate their capability to undergo germinal vesicle break down (GVBD) and reach the metaphase II (MII) stage. We found that during the first 28 days of culture, a significant number of oocytes within the ovarian explants reached nearly full growth and formed preantral follicle-like structures with the surrounding somatic cells. GSH level and *Cx37* expression in the oocytes within the explants were indicative of proper developmental conditions. Moreover, the imprinting of *Igf2r* and *Peg3* genes in these oocytes was correctly established. Further culture onto PAGCs in the presence of ActA allowed about 16% of the oocytes to undergo GVBD, among which 17% reached the MII stage during the final 16–18 hr maturation culture. These MII oocytes showed normal spindle and chromosome assembly and a correct ERK1/2 activity. About 35% of the in vitro matured oocytes were fertilized and 53.44% of them were able to reach the 2-cell stage. Finally, around 7% of the 2-cell embryos developed to the morula/blastocyst stage.

## Introduction

In mammalian females, oocytes originate from embryonic precursors termed the primordial germ cells (PGCs) [1]–[3]. In mice, PGC precursors arise in the proximal epiblast at around 6.25 days *post coitum* (dpc), and then move into the extraembryonic mesoderm at the base of the allantois where by 7.25 dpc they are specified as PGCs [4]–[5]. Between 7.5 to 8.5 dpc, PGCs move caudally from the extraembryonic mesoderm into the hindgut endoderm from where they migrate through the dorsal mesentery of the hindgut into the gonadal ridges from 9.5 to 11.5 dpc. Within the developing ovaries, PGCs continue to proliferate and finally around 13.5 dpc enter meiosis becoming primary oocytes [6]–[9]. Most oocytes have entered meiosis by 14.5 dpc, and become arrested at the diplotene stage of the first meiosis around birth (19.5 dpc -1-2 days *post partum*, dpp) [7], [10]. At this stage, oocytes are individually surrounded by pregranulosa cells to form primordial follicles [2], [11], [12].

Oocyte growth and maturation are strictly dependent on establishing functional communications with the surrounding granulosa cells through gap junctions and reciprocal interactions mediated by paracrine and endocrine signals. These processes are accurately regulated by numerous growth factors and hormones (for a review, see [13], [14]).The complex regulatory mechanisms controlling the early stages of oogenesis in mammals are only partially understood because of the difficulty to differentiate oocytes from PGCs *in vitro* [15]. A real challenge is to establish an *in vitro* culture system for studying oogenesis starting from PGCs and ending in producing mature oocytes [16]. Moreover, such a system could potentially provide an unlimited source of oocytes for biomedical application and invaluable for establishing the conditions to generate oocytes from stem cells, one of the major challenges of the present biology of reproduction [15], [17]. Several attempts have been made to reproduce early mouse oogenesis *in vitro* [18]–[30]. Oocytes derived from newborn mouse ovaries can undergo an apparent normal development *in vitro*, and the matured oocytes support the development to term to some extent [7], [18], [22]. In contrast, oocytes generated from PGCs or premeiotic germ cells *in vitro* remained arrested at the prophase of the first meiotic division and failed to complete maturation [7], [8], [19], [20], [23], [31]. Only combining *in vivo* ectopic transplantation of embryonic ovaries and *in vitro* culture of follicles, mature oocytes have been obtained from premeiotic 12.5 dpc germ cells by others and us [25], [26], [32], [33].

Activin A (ActA), a member of the transforming growth factor beta (TGFβ) super-family produced in the ovary as well in a variety of other organs (for a review, see [34]), is an important modulator of preantral follicle development in various species including humans [35]–[44]. Beside as local regulator of folliculogenesis, ActA is able to directly stimulate FSH synthesis and secretion, and to promote the release of the gonadotrophin-releasing hormone (GnRH) [43]. ActA can also stimulate the increase of FSH and LH receptors in granulosa cells, and plays roles in progesterone production and aromatase induction [41], [43]. Thus, granulosa cells are likely to be the main source of the paracrine factors, and are crucial for oocyte maturation.

The purpose of this study was to establish a simple method for obtaining mature oocytes from premeiotic female germ cells entirely *in vitro*. We found that the presence of ActA during the culture of explants of embryonic ovaries and the subsequent coculture of the growing oocytes generated within the explants onto granulosa cell monolayers were crucial for obtaining significant number of fertilizable oocytes that are able to develop to morula/blastocyst stages.

## Materials and Methods

### Animals

All procedures described in the present study were reviewed and approved by the Ethical Committee of Qingdao Agricultural University (No. 20090617). If not otherwise indicated, for easier recognition of the exogenous oocytes in the oocyte-preantral granulosa cells (PAGCs) cocultured experiments, transgenic mice carrying the Enhanced Green Fluorescent Protein driven by chicken beta-actin promoter and CMV intermediate early enhancer (EGFP transgenic mice [45] and CD-1 mice (Vital River, Beijing, China) were used (female: 6–8 weeks; male: 10–12 weeks) and maintained on a 12:12-h light/dark cycle (lights off at 20:00). The appearance of copulation plug was designated as 0.5 dpc.

### Isolation of Oocytes from Adult and Prepubertal Mice and in vitro Culture of Immature Oocytes and PAGCs

**Figure 1:**
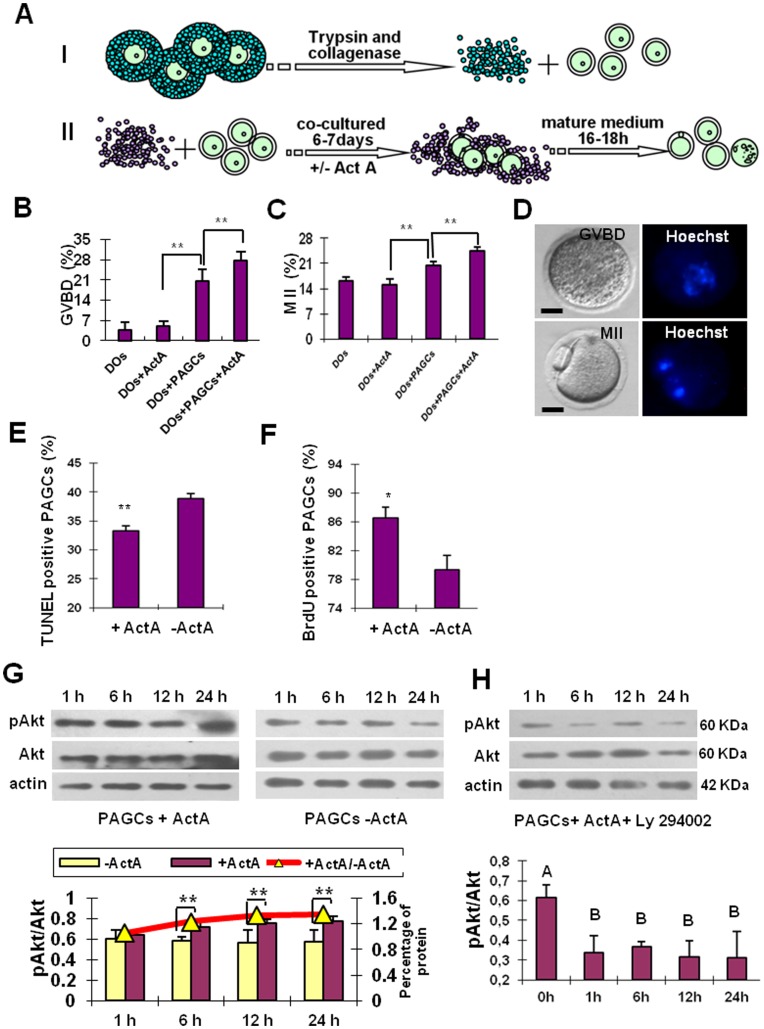
*In vitro* culture system of immature oocytes from preantral follicles. (A) Schematic drawing of the main isolation and culture procedures. Oocytes (diameter 55–65 µm) were collected from 12–14-day-old EGFP transgenic mouse and preantral granulosa cells (PAGCs) were collected from 12–14-day-old mouse. PAGCs were cultured as described in M&M, denuded oocytes were divided into four groups: oocytes cocultured with PAGCs in the presence (DOs+PAGCs+ActA) or absence of 100 ng/ml ActA (DOs+PAGCs) for 6–7 days, and oocytes cultured alone in the presence (DOs+ActA) or absence of ActA (DOs) for the same period. Finally, GV oocytes were collected and matured in the *in vitro* maturation medium (IVM) for 16–18 h. (**B–C**) *In vitro* maturation of the oocytes of the four experimental groups as indicated by percents of GVBD and MII oocytes. Culture onto PAGCs results in a significant increase in the number of both GVBD and MII oocytes; ActA significantly improves both maturation capabilities. (**D**) An example of GVBD and MII oocytes observed under Nikon optics (left) and after DNA staining with Hoechst (right). (**E**) ActA decreases the number of apoptotic PAGCs cultured *in vitro* for 7 days as evaluated by the TUNEL staining. (**F**) Incubation for two days in the presence of ActA increases the number of BrdU positive PAGCs *in vitro*. (**G**) ActA increases phospho-Akt in PAGCs cultured *in vitro* for 1, 6, 12 and 24 h (immunoblotting and densitometric analysis). (**H**) The PI3K-specific inhibitor, LY294002, inhibits Akt phosphorylation at a concentration of 25 µM (immunoblotting and densitometric analysis). *P<0.05; **P<0.01.

Immature oocytes were isolated from 12–14-day-old EGFP transgenic mice, and PAGCs were isolated from 12–14-day-old prepubertal wild type mice as follows (Fig. 1A). Briefly, follicles were released by puncturing the ovaries in M2 medium [46]. They were then incubated for 15 minutes at 37°C in 0.25% trypsin (Gibco-BRL, Carlsbad, CA), 0.2% collagenase IV (Gibco-BRL) plus 0.02% DNase-I (Sigma) solution, and finally repeatedly pipetted with a drawn pipette. After adding culture medium containing α-minimal essential medium (α-MEM) and 10% heating-inactivated fetal calf serum (FCS) (Gibco-BRL), the cell suspension was resuspended in 100 µl of pre-warmed culture medium. Denuded oocytes (DO) and PAGCs were separated and used for subsequent experiments as indicated.

PAGCs obtained from 20–30 follicles were transferred to 100 µl of culture medium (IVC) in a 6 cm culture dish (BD Biosciences, Bedford, MA, USA) covered with 6 ml of mineral oil (Sigma). The culture medium was composed of α-MEM supplemented with 10% FCS, 1% insulin-transferrin-selenium mix (ITS-mix; 1 mg/ml, 0.55 mg/ml and 5 ng/ml) (Gibco-BRL), 10 mIU/ml follicle-stimulating hormone (FSH; Sigma), 5 ng/ml recombinant epidermal growth factor (rEGF; Sigma), 0.23 mM pyruvic acid, 100 IU/ml penicillin G and 100 mg/ml streptomycin sulfate. Cell culture was carried out at 37°C in a humidified atmosphere supplemented with 5% CO_2_. Within 24 h, the granulosa cells spread out to form a complete monolayer. At this time 20 oocytes were seeded in each well (see below). Half of the medium was changed on the third day of culture.

Denuded oocytes were divided into four groups: oocytes alone (denuded oocytes, DOs), oocytes alone with 100 ng/ml ActA [45] (DOs+ActA), oocytes cocultured onto PAGCs (DOs+PAGCs) and oocytes cocultured onto PAGCs in the presence of ActA (DOs+ PAGCs+ ActA). All cultures were carried in drops of 100 µl of medium in 6 cm dishes covered with 6 ml of mineral oil. The cultures were carried out for 6–7 days at 37°C in a humidified atmosphere supplemented with 5% CO_2_.

### Histology and Measurement of Follicles

Phosphate buffered solution (PBS) or ActA in PBS at the dose of 60 µg/kg (Sigma, St. Louis, MO, USA) was delivered to mice after 10 dpp by daily intraperitoneal injection. Ovaries were collected after the injection for 2, 4 and 6 days, and then fixed in Bouin’s fluid for 24 h and embedded in paraffin. Serial sections of 5 µm in thickness were stained with haematoxylin and eosin (Sigma, St. Louis, MO, USA). In order to quantitatively evaluate the number of primordial, primary and secondary follicles, slides from each ovary were arranged according to the order and every fifth section was marked for analysis. Follicles containing an intact oocyte with a nucleus and a single layer of flat granulosa cells were classified as primordial follicles. Follicles containing of an intact oocyte with a nucleus and a single layer of cuboidal granulosa cells were termed as primary follicles. Follicles containing an intact oocyte with a nucleus and two layers of granulosa cells were scored as early secondary follicles. Follicles containing an intact oocyte with a nucleus and more than two layers of granulosa cells were scored as secondary follicles. Similarly, in order to estimate the total number of follicles in each ovary, the total number of primordial, primary, secondary and antral follicles in the marked sections was multiplied by five, taking into account the fact that every fifth section was used in the analysis.

### Isolation and Culture of 12.5 dpc Female Mouse Gonads

Fetal mouse gonads obtained from 12.5 dpc EGFP transgenic fetuses without mesonephros were divided into two pieces and cultured in 600 µl of medium composed of α-MEM supplemented with 10% FCS, 0.23 mM pyruvic acid, 10 mIU/ml FSH, 100 mIU/ml penicillin G and 100 mg/ml streptomycin sulfate in the presence and absence of 100 ng/ml ActA [24], [25]. In a 24-well plate (BD Biosciences) at 37°C in a modular incubation chamber (Billups Rothenberg, Del Mar, CA, USA) infused with a gas mixture of 5% CO_2_ and air. On the next day, the medium was increased to 900 µl, and the depleted medium was refreshed by exchanging 300 µl fresh medium every other day [25]. Unless indicated the culture was carried out for 28 days. When indicated, the oocytes formed within the ovarian tissue explants were isolated by incubating the tissues for 10–15 minutes in EDTA/trypsin and picked up with a drawn pipette for analysis or for further *in vitro* culture as described in the Results.

#### Maturation culture of the oocytes

Oocytes of all experimental groups were finally isolated and matured in 25 µl drops under mineral oil of maturation medium composed of α-MEM supplemented with 10% FCS, 100 mIU/ml FSH, 1 ng/ml rEGF, 0.23 mM pyruvic acid, 100 U/ml penicillin G and 100 mg/ml streptomycin sulfate. Cultures were carried out for 16–18 h at 37°C in a humidified atmosphere supplemented with 5% CO_2_. At the end of the culture, the oocytes were examined under a stereomicroscope to determine the percentage that had undergone germinal vesicle breakdown (GVBD) and produced a polar body (MII stage). As a control cumulus-enclosed oocytes (COCs) were isolated, from the ovaries of 21–22-day-old CD-1 mice by puncturing the largest antral follicles with a needle, and these GV oocytes were matured and fertilized in vitro (see below).

### RNA Extraction, cDNA Synthesis and Quantitative PCR in Oocytes and PAGCs

Total RNA from 400 oocytes or 10^4^ PAGCs was extracted by using an RNAprep pure Micro Kit (Tiangen, Beijing, China) according to the manufacturer’s instructions. The RNA was resuspended in 11 µl of nuclease-free water. Preantral granulosa cell cDNA was synthesized by using Superscript II reverse transcriptase (Invitrogen). Briefly, 20 µl of reaction mixture containing 11 µl of total RNA, 4 µl of 5× RT buffer, 1 µl of Oligo (dT) 20 primer, 2 µl of dNTPs, 1 µl of RNase inhibitor and 1 µl of Rever TraAce was allowed to react as specifically designed by the manufacturer. The real-time PCR was carried out using the SYBR® Premix Ex Taq™ kit (TaKaRa, Dalian, China) and performed on a Roche LightCycler 480 II real-time PCR instrument (F. Hoffmann-La Roche, Ltd) by using the standard curve method with *β-actin* as the reference gene. The primers used to amplify *Cx37, Bcl-2 and Bax* and *β-actin* were listed in Table S1. Amplification reactions were performed in 25 µl of reaction mixture containing 2 µl of cDNA, 12.5 µl of SYBR® Premix Ex Taq™ (2×) (TaKaRa), 9.5 µl of RNase-free water and 1 µl of forward and reverse specific primers (5 µM) for each gene according to manufacturer’s protocol. The average value and SEM were calculated from triplicate measurements, and the relative amount of gene expression for each sample was plotted. All data were expressed as Mean ± SEM [24], [25], [31], [47]–[48].

### DNA Isolation, Bisulfite Sequencing and DNA Methylation Analysis in Oocytes

DNA was isolated by using a Proteinase K/SDS method, as described previously [49]. Briefly, 300–500 oocytes in each sample were resuspended in 18 µl of lysis solution containing 2 µg *E. coli* tRNA, 1 mM SDS, and 280 µg/ml proteinase K. The samples were sequentially incubated at 37°C for 30–90 min, and at 98°C for 15 min. The isolated DNA was treated with sodium bisulfite from a Methylamp™ DNA modification kit (Epigentek, USA) according to the manufacturer’s instructions. PCR reactions for *Igf2r* (insulin like growth factor 2 receptor), *Peg3* (paternal expressed gene 3) and *H19* genes were carried out by using bisulfite-treated DNA template and the primers specific to the converted DNA. The bisulfite converted DNA was amplified by nested PCR. The primers for the amplification of *Igf2r*, *Peg3* and *H19* were shown in Table S2. The DNA fragments separated by electrophoresis in 1% agarose gel were excised and purified with the Wizard SV gel and PCR clean-up system (Promega). The purified DNA was cloned into a pDM18-T vector (TaKaRa, Dalian, China) according to the manufacturer’s instructions. The positive clones were screened by aminobenzylpenicillin selection and the insert was sequenced at Union Gene (Shanghai, China)[48], [50]–[52].

### Immunoblotting Blot Analysis

The proteins from 100 oocytes or 10^5^ granulosa cell were extracted by vortexing the samples in RIPA lysis solution (Beyotime) for 30 min before adding appropriate volume of SDS and boiling for 5 min. Total protein was separated by SDS-PAGE for 50 min at 100 V followed by 1.5 h at 120 V, respectively. The separated proteins were transferred onto nitrocellulose membrane and then blocked in TBST buffer containing 10% BSA (Sigma) for 4 h at room temperature. The blot was incubated with rabbit anti-phospho-Akt (Ser 473) antibody (Abcam, Hong Kong) at the dilution ratio of 1∶500 in TBST buffer at 4°C for 2 h. After washing with TBST buffer for 3 times with 5 min each time, the membrane was incubated with horseradish peroxidase (HRP)-conjugated goat anti-rabbit IgG (Beyotime) at the dilution ratio of 1∶1000 in TBST at 37°C for 1 h. The membrane was washed with TBST buffer for three times and then processed by using the enhanced chemiluminescence (ECL) detection system. The membrane was washed to strip off bound antibody, and re-probed with polyclonal rabbit anti-Akt antibody (Beyotime) at the dilution ratio of 1∶800, and then incubated with HRP-labeled goat anti-rabbit IgG at the dilution ratio of 1∶1000. Similarly, the blot was cut according to 68-kDa molecular weight marker and then incubated with murine anti-p-ERK1/2 antibody (Santa Cruz, USA) at the dilution of 1∶500, and re-probed with polyclonal rabbit anti-ERK2 antibody (Santa Cruz) at the dilution of 1∶300. The band intensity was quantified by using β-actin (Abcam) as an internal control and IPWIN software was used for measuring the intensity of the bands. All experiments were repeated at least 3 times [53]–[55].

### Analysis of the Oocyte Spindle

Oocyte zona pellucida (ZP) was removed with acidic M2 and zona-free oocytes were fixed in 4% paraformaldehyde for 30 min. and incubated with α-tubulin antibody (Sigma, USA) for 2 h. Nucleus was stained with propidium iodide (PI) for 15 min. Finally, oocytes were mounted on the slides with the DABCO antifade mounting agent and covered by a coverslip [48]. The oocytes were examined with a confocal laser-scanning microscope (Zeiss LSM 710 META, Germany).

### Fertilization and *in vitro* Culture of Embryos

The caudal epididymis was removed from 10- to 12-week-old CD1 male mice and placed in 1 ml of a mutant potassium simplex optimized medium (mKSOM) supplemented with 0.4% (w/v) BSA in a sperm dispersion dish. The dish was placed in an incubator supplied with 5% CO_2_ for 20 min to allow the sperm to disperse. Totally 10 µl of sperm suspension was added to 90 µl of mixture of mKSOM with BSA in a fertilization dish. Capacitation was allowed to proceed for 45–60 min at 37°C in the incubator.

Oocytes were transferred to the fertilization droplet and incubated at 37°C in a modular incubation infused with a gas mixture of 5% CO2 and air for 4 h. Ten inseminated oocytes were incubated for 48 h in a 20 µl drop of mixture of KSOM- BSA to detect pronuclear formation and the capability to develop to 2-cell stage. Finally, the medium was replaced with 20 µl of mixture of mKSOM with BSA for an additional 48 h culture for morula/blastocyst development [24], [25].

### Statistical Analysis

Statistical analysis was performed by using SAS 9.1 (SAS Institute Inc, Cary, NC) and Prism 4 (GraphPad Software Inc., San Diego, CA) softwares. The results were analyzed by variance analysis (ANOVA), and the difference was subjected to Tukey’s test. Normalized cycle threshold (Ct) values from real-time PCR were analyzed by Kruskal-Wallis test, and Dunn multiple comparison tests were performed among different groups. A significant difference was considered at *P*<0.05 for all tests.

## Results

### Establishment of *in vitro* Maturation Culture System for Immature Oocytes from Prepubertal Ovaries

In a recent study, we found that it was possible to obtain growing mouse oocytes *in vitro* from 12.5 dpc premeiotic female germ cells, but these oocytes were unable to complete the growing phase and thus fail to reach maturation [16]. The maximum diameter and the general morphological features of these oocytes after 21 days of culture were similar to those of 55–65 µm oocytes from 12–14 dpp ovaries. We reasoned that *in vitro* conditions suitable for promoting maturation of *in vivo* immature oocytes could also be proper for supporting maturation of *in vitro* generated oocytes. We decided to use preantral granulosa cell (PAGC) monolayers and activin A (ActA) as potential inducers of oocyte maturation (see Introduction). Oocytes were isolated from 12–14 dpp ovaries and cultured for 6–7 days under four different conditions: DOs, DOs+ActA, DOs+PAGCs and DOs+PAGCs+ActA (Fig. 1A, Fig. S1 and Supporting Information S1).

In order to detect the *in vitro* meiotic maturation ability of the oocytes cultured under different conditions described above, germinal vesicle breakdown (GVBD) and polar body emission (the metaphase II, MII) were evaluated (see Materials and Methods) (Fig. 1B–D). In the DOs+PAGCs+ActA group, the percents of GVBD and MII oocytes were 27.60±1.20% and 24.50±1.09% (n = 1120), respectively, significantly higher than those in the DO+ PAGCs group (20.5±1.01% and 20.5±0.95% (n = 1233), respectively) and in the groups without PAGCs (around 7% and 14%, respectively) (*P*<0.05 or *P*<0.01). As note, in these latter groups the capability of oocytes to undergo GVBD and reach MII was not influenced by the presence of ActA (Fig. 1B–C). Under the same culture conditions, 95.7% (111/116) of the oocytes from 21–22-day-old females underwent GVBD and 86.5% (96/111) of them progressed to the MII stage.

In order to clarify the action of ActA on the granulosa cells, we investigated the effect of the growth factor on the proliferation and apoptosis of the PAGCs (Supporting Information S2 and S3). Using the BrdU incorporation assay, we found that ActA significantly promoted the proliferation of PAGCs after 2 days of stimulation in culture (86.47±1.55% vs 79.33±2.07%; *P*<0.05) (Fig. 1E and Fig. S2A). Furthermore, ActA caused a significant reduction of PAGC apoptosis after 7 days of culture as evaluated by the TUNEL assay (38.86±0.92% vs 33.34±0.87%; *P*<0.01) (Fig. 1F and Fig. S2B).

To clarify the molecular pathways stimulated by ActA in PAGCs, the phosphorylation of Akt in these cells was assessed. The levels of Akt and phospho-Akt in PAGCs cultured *in vitro* for 1, 6, 12 and 24 h were detected (Fig. 1G). The results showed that the ratio of pAkt/Akt was increased in PAGCs cultured with ActA during 1–24 h. The phosphorylation of Akt was significantly inhibited by pre-treatment with the PI3K-specific inhibitor LY294002 (Fig. 1H). Thus these results indicate that ActA promotes the phosphorylation of Akt in PAGCs *via* the canonical PI3K signaling pathway.

### ActA Promotes Follicle Development *in vivo*

**Figure 2:**
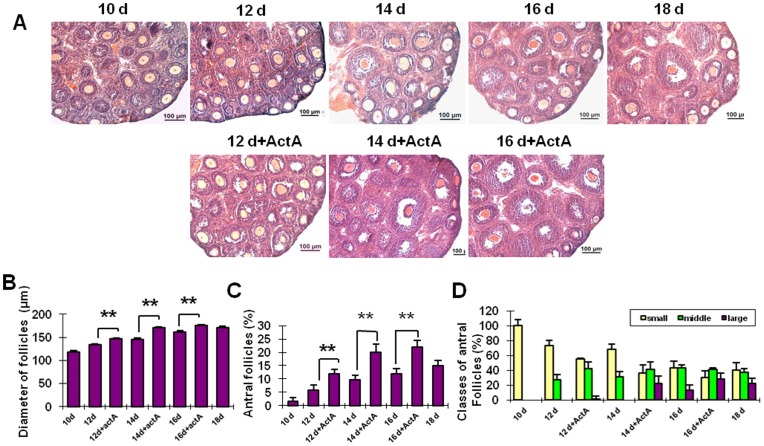
Injection of ActA promotes follicologenesis. Injection of ActA for 2, 4 and 6 days results in a significant increase in the diameter of follicles (**A, B**) and the percent of antral follicles (**C**) when compared to the control group. Antral follicles were divided into three groups: small (<140 µm), medium (140–180 µm) and large (>180 µm). The proportion of antral follicles in each group is shown in D. Scale bars: 100 µm. *P<0.05; **P<0.01.

In order to explore the impact of ActA on follicular development *in vivo*, ActA was injected into 10 dpp female mice at the dose of 60 µg/kg/day for 6 consecutive days (Fig. 2A and Fig. S3). After ActA injection for 2, 4 and 6 days, the follicular diameters were 147.60±1.31, 170.22±2.41 and 175.39±2.15 µm, respectively, significantly higher than those in the control group (133.36±2.17, 145.31±2.85 and 161.48±3.22 µm) (Fig. 2 B) (*P*<0.01). In addition, in order to evaluate the effect of ActA on the follicular dynamics, the percent of antral follicles was also evaluated. As shown in Fig. 2C, the percentages of small antral follicles were 1.50±1.38%, 5.80±1.82%, 9.80±1.61% and 12.03±1.77% at the postnatal days of 10, 12, 14 and 16, respectively in control groups. However, the populations of small antral follicles in the presence of ActA were 11.70±1.59%, 20.40±3.14% and 22.00±2.62% at the postnatal days 12, 14 and 16, significantly higher than those of the control groups (*P* < 0.01). In order to further analyze the follicular development, the antral follicles were divided into three groups: small follicles (<150 µm), medium follicles (150–180 µm) and large follicles (>180 µm). As shown in Fig. 2D, the degree of follicular growth was significantly increased following ActA administration.

### ActA Promotes the Survival and Growth of Oocytes Generated from Premeiotic Germ Cells *in vitro*

Fetal ovaries were isolated from 12.5 dpc fetuses. Each ovary without mesonephros was then divided in two pieces and cultured in the presence or absence of 100 ng/ml ActA. Morphological examination of the explants revealed that the oocytes in the ovarian tissues cultured with ActA were more numerous and exhibited a higher growth rate than the group without ActA. In the presence of ActA, structures resembling primordial/primary and preantral secondary follicle were also observed from 12 days of culture onwards (Fig. S4).

**Figure 3:**
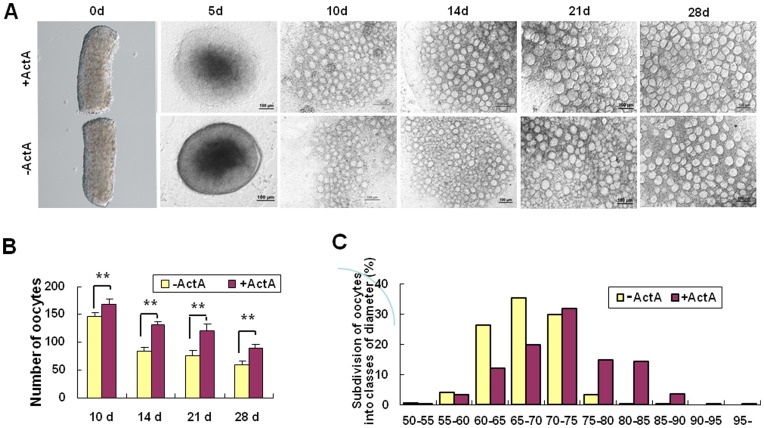
*In vitro* culture of embryonic ovary explants. (**A**) Pieces of ovaries from 12.5 dpc mouse embryos were cultured *in vitro* in the presence or absence of ActA for up to 28 days. (**B**) Number of oocytes scored during the explant culture *in vitro* in the presence or absence of ActA at 10, 14, 21 and 28 days. (**C**) Diameters of oocytes in the presence or absence of ActA for 28 days. IVC = *In vitro* culture medium; IVM = *In vitro* maturation medium. *P<0.05; **P<0.01.

As shown in Fig. 3A and Fig. S5, at the day 10 of culture the number of oocytes in the explants cultured in the presence of ActA was 168.52±7.13, significantly higher than that in the explants without ActA (146.08±8.77) (*P*<0.01). Similarly, at the day 28 of culture, the number of oocytes in the experimental group with ActA was 89.63±6.38 and 59.52±7.14 in the group without ActA (*P*<0.01). After 28 days of culture, the percent of the oocytes with a diameter between 75–80 µm was significantly higher in the explants with ActA. Moreover, oocytes with diameter greater than 85 µm were only observed in the presence of ActA (Fig. 3B–C).

At 28 days of culture, the increased number of oocytes generated in the presence of ActA was associated to reduced apoptosis (235/826, 28.45±0.55% TUNEL positive oocytes *vs* 34.41±1.33%, 96/279, *P*<0.01, Fig. S6A). In line with the apoptosis evaluation, we found that *Bcl-2* and *Bax* transcripts were increased and decreased, respectively, in oocytes generated in the presence of ActA in comparison to that formed in the absence of the growth factor (Fig. S6B). Finally, the levels of GSH and of the *Cx37* transcripts measured in oocytes >50 µm in diameter were much higher in the presence of ActA (GSH, 17.42±3.75 µM *vs* 5.53±0.76 µM, *P*<0.01, Fig. D6C) (*Cx37,* Fig. S6D).

### ActA Promotes the Methylation of Oocytes Derived from Premeiotic Germ Cells *in vitro*

**Figure 4:**
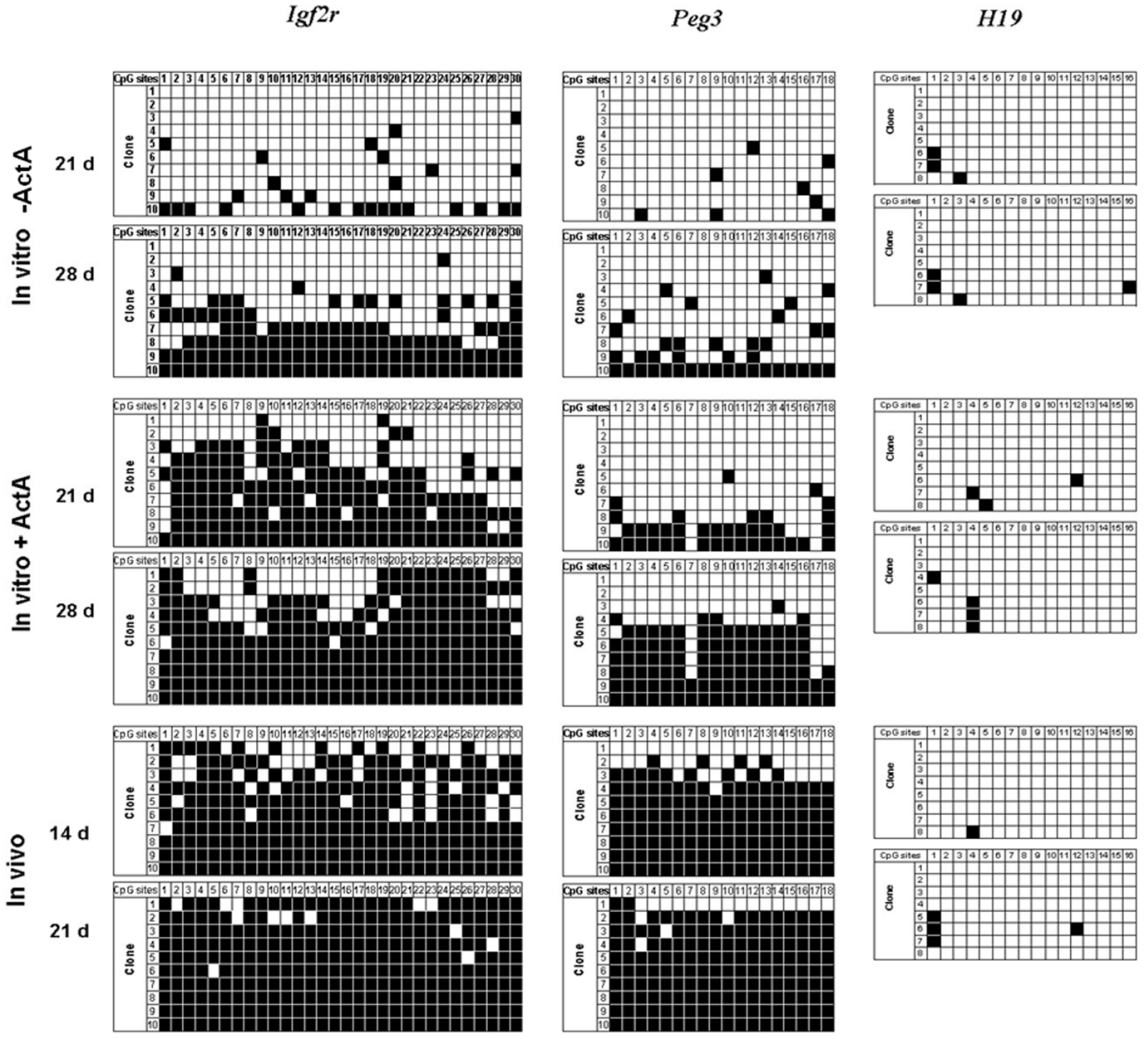
Establishment of the gene imprinting in the oocytes generated *in vitro*. Bisulfite sequencing analysis of DMR in maternally imprinted *Igf2r*, *Peg3* and *H19* in the oocytes derived from 12.5 dpc ovaries cultured *in vitro* for 21 and 28 days in the presence or absence of ActA, as well as the oocytes from 14 and 21 dpp ovaries. Individual lines, clones sequenced; circles, CpG sites within the analyzed regions; filled circles, methylated cytosines; open circles, unmethylated cytosines.

To determine whether DNA methylation of imprinted genes in the oocytes from premeiotic germ cells was completed during *in vitro* culture, the oocytes cultured within the ovarian explants for 21 and 28 days with or without ActA were collected, and the differentially methylated regions (DMRs) of the maternal imprinted genes *Igf2r* and *Peg3* were examined. As shown in Fig. 4, the percentage of methylated CpG sites in the *Igf2r* and *Peg3* DMRs were 61.71% and 20.02% respectively in the oocytes in the presence of ActA *in vitro* for 21 days, and increased to 81.32% and 46.10% after culture for 28 days, respectively. These values were much, higher than those in oocytes cultured in the absence of ActA (*Igf2r*, 9.31 to 42.72%; *Peg3*, 4.44 to 21.72%, at 21 and 28 days, respectively). *H19*, a paternal imprinted gene, was also analyzed as a control. We found that *H19* gene maintained its correct unmethylated status during 21 and 28 days of culture with or without ActA.

### ActA Promotes Meiotic Maturation of Oocytes Generated from Premeiotic Germ Cells *in vitro*

The oocytes isolated from the explants after 28 days of culture with or without ActA and soon subjected to the maturation assay were unable to undergo GVBD regardless of their diameter (GVBD 0/551).

**Figure 5:**
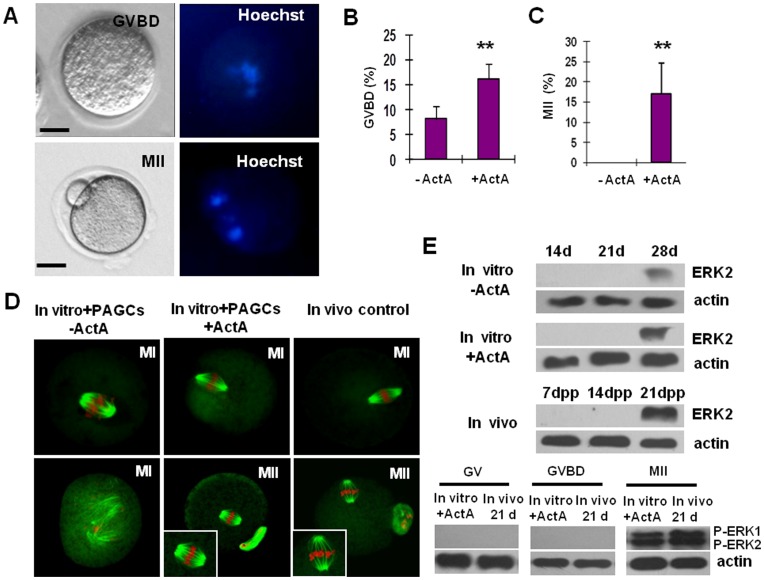
Characterization of the maturation status of the oocytes generated *in vitro* from 12.5 dpc embryonic ovaries. (**A**) An example of GVDB and MII oocytes observed under Nikon optics (left) and after DNA staining with Hoechst (right). (**B, C**) Percents of GVDB and MII oocytes generated within the ovarian explants after 28 days of culture in the presence of ActA and cocultured onto PAGCs for 7 days in the presence or absence of ActA. (**D**) MI and MII spindle morphology in oocytes. Oocytes generated *in vitro* from premeiotic germ cells (In vitro+PAGCs+ActA) show normal MI spindle, MII spindle and chromosome assembly as compared to those of control oocytes isolated from ovaries of 21–22-day-old mice (In vivo control). In contrast, oocyte generated *in vitro* as described above but cocultured onto PAGCs in the absence of ActA (In vitro+PAGCs-ActA) show anomalous MI spindle and chromosome misalignment. (**E**) Phosphorylation (activation) of ERK1/2 in oocytes as detected by immunoblotting. Expression of ERK2 protein was analyzed in oocytes obtained from ovarian explants at 14, 21 and 28 days of culture + or – ActA and from 7, 14 and 21 dpp ovaries; only 28 day oocytes in vitro and 21 dpp oocytes in vivo showed detectable ERK2 expression. Phosphorylation of ERK1/2 (pERK1/ERK2) was analyzed in GV, GVDB and MII oocytes generated *in vitro* (28 days within ovary explants+ActA and 7 days onto PAGCs+ActA) or isolated from ovaries of adult females (21 d); only *in vitro* and *in vivo* MII oocytes showed pERK1/ERK2. *P<0.05; **P<0.01.

In order to promote the meiotic maturation, oocytes with a diameter >50 µm after culture in the presence of ActA for 28 days were isolated and further cocultured with PAGCs for 6–7 days in the presence or absence of ActA (Fig. S5). During this period, no significant increase in the oocyte diameters was observed (data no shown). At the end of this period, the capability of the oocytes to undergo GVBD and reach MII in IVM was evaluated. The results showed that 95/581 (16.35%) of the oocytes cocultured onto PAGCs with ActA underwent GVBD, amont which 17/95 (17.89%) reached MII stages. In contrast, only 15/183 (8.20%) of the oocytes cocultured onto PAGCs without ActA underwent GVBD and none of them (0/15) were able to reach MII (*P*<0.01) (Fig. 5A–C). Oocytes cultured with PAGCs in the presence of ActA showed normal MI and MII spindles. In contrast, although part of the oocytes cocultured with PAGCs in the absence of ActA were able to undergo GVBD, they failed to form typical spindle structure and the chromosomes were misaligned (Fig. 5D).

The meiotic maturation of oocytes is regulated by a cascade of protein phosphorylation/de-phosporylation events. Two isoforms of MAP kinases, ERK1 (p44) and ERK2 (p42), appear to play a pivotal role in this orchestration [56], [57]. For this reason, ERK1/2 phosphorylation during the final *in vitro* maturation of the *in vitro* generated oocytes was also investigated by immunoblotting. The results showed that after 16–18 hr in IVM, ERKs were activated in MII oocytes derived from ovarian explants and PAGC cocultures in the presence of ActA and in control MII oocytes obtained from adult ovaries (21–22 dpp) as well. On the contrary, ERKs remained inactive in GV and GVBD oocytes generated in the presence of ActA and subcultured onto PAGCs without ActA (Fig. 5E).

### *In vitr*o Generated Oocytes from Premeiotic Germ Cells Support Fertilization and Morula-blastocyst Development

**Figure 6:**
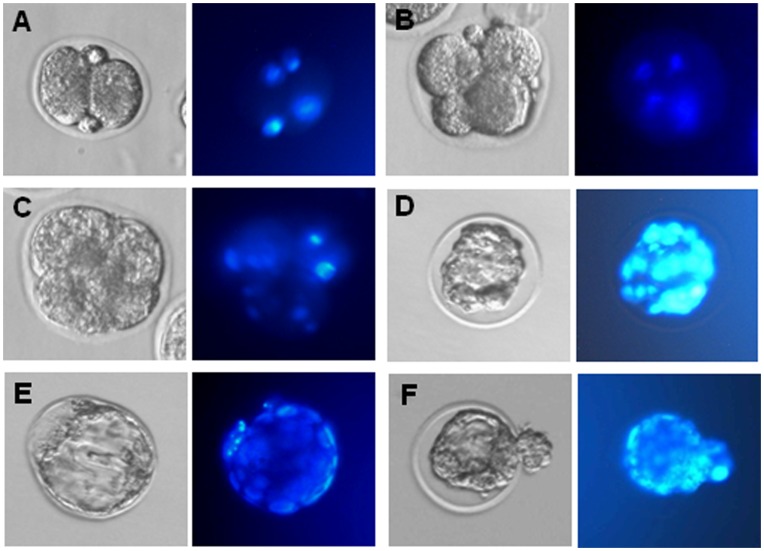
Oocytes generated *in vitro* in the presence of ActA can be fertilized and fertilized eggs develop to morula and blastocyst stages. (**A–E**) Example of 2-cell embryo, 4-cell embryo, morula and blastocyst developed from oocytes generated *in vitro* in the presence of ActA. Left, phase contrast microscopy observations; right, Hoechst staining of the cell nuclei.

In order to assess the developmental capacity of the oocytes produced and matured as reported above in the presence of ActA, they were subjected to *in vitro* fertilization (Fig. 6). 189 of 533 (35.46%) oocytes (GVBD and MII stage oocytes) were fertilized as shown by the presence of two pronuclei, among which 53.44% (101/189) reached 2-cell stage. Finally, 6.93% 2-cell embryos (7/101) developed to the morula/blastocyst stage (Table 1). As a control 90.6% (87/96) MII oocytes obtained from 21–22 days old mice were fertilized, and 95.4% (83/87) of fertilized eggs developed to 2-cell embryos, among which 95.2% (79/83) reached morula/blastocysts.

**Table 1:** Development of embryos produced by the oocytes from pre-meiotic germ cells in vitro.

	oocytes	Fertilized	2-cell	4-cell	8-cell	Morula/Blastocyst
*Number*	533	189	101	42	26	7
*Percentage*	–	35.46	53.44	41.58	25.74	6.93

## Discussion

In the present paper, we report for the first time the production of significant numbers of mature and fertilizable mouse oocytes exclusively through *in vitro* culture. We achieved this result essentially on the basis of two key findings. First, previous observations by others and us have shown that immature mouse GV oocytes can be obtained from premeiotic germ cells after a prolonged period of *in vitro* culture from explants of embryonic ovarian tissues [8], [15], [58]–[61]. Second, the results obtained in the present paper that immature mouse GV oocytes can be matured *in vitro* following a 7 day coculture onto PAGCs in the presence of 100 ng/ml ActA. In particular, in the first series of experiments, we found that PAGCs and ActA cooperated to ensure the growth and maturation of immature oocytes *in vitro*. Granulosa cells can provide nutrients and signal molecules crucial for oogenesis [62]. ActA belongs to the TGF-β superfamily including inhibins, mullerian inhibitor substance (MIS) and bone morphogenetic proteins (BMP), which are autocrine/paracrine intraovarian regulators (see Introduction) [63]. In line with our present data, previous results showed that ActA was able to increase the developmental competence of bovine and human oocytes [40], [64], [65].

In the present paper, we obtained also evidence that while PAGCs favored mainly the *in vitro* survival of the immature oocytes by reducing apoptosis, ActA exerted a more complex effect on the maturation of such oocytes. In fact, we found that ActA favored both the accumulation of GSH and presumably oocyte-granulosa cell communications (increasing level of *Cx37* transcripts). It is well known that high level of GSH is considered a reliable marker of oocyte cytoplasmic maturation [64] and that gap junctions between the oocyte and the surrounding granulosa cells are required for proper oocyte growth and maturation. Cx37 is present in gap junctions between the oocyte and surrounding cumulus cells and has been localized at the surface of the oocytes. The gap protein Cx32, Cx43, and Cx45 have been localized between granulosa cells in mouse ovarian follicles. Cx43 and Cx37 are thought to play more critical roles in ovarian function because the absence of either connexins causes a loss of oocyte-granulosa cell coupling and disruption of folliculogenesis [66]–[71].

Our data also showed that the ActA effects on the immature oocytes were dependent on the presence of PAGCs. The proliferation promotion effect and anti-apoptotic effect of ActA in the presence of the PAGCs that we demonstrated here are in line with its complex role as local regulator of folliculogenesis reported in several species [65], [70]. It must be pointed out, however, that the growth and the capability to resume meiosis and reach the MII stage of the immature oocytes cultured onto PAGCs plus ActA are still significantly lower than those of oocytes of equivalent *in vivo* chronological age (21–22 dpp). Thus, these data indicate that co-culture with ActA and onto PAGCs can only partly reproduce the complex ovarian environment necessary for complete oocyte maturation.

Most importantly, in the second series of experiments, we were able to produce significant number of mature and fertilizable oocytes by adding ActA to the medium used for the embryonic ovarian explants culture and maintaining this growth factor during the subsequent coculture onto PAGCs. ActA was able to markedly increase the number and the growing rate of oocytes within the explants. Moreover, most of the oocytes cultured in the presence of ActA became surrounded by one or more rings of flatted or cuboidal granulosa cells resembling primordial and primary/early secondary follicles, respectively (Fig. S4A–B). This latter result is in line with previous results showing that ActA is able to organize two dimensional rat follicles from monolayers cultures of granulosa cells and oocytes *in vitro* [42]. The observations that apoptosis evaluated by TUNEL and *Bax* mRNA was reduced in oocytes in the presence of ActA while *Bcl2* mRNA level was increased, suggest that the increased number of oocytes was mainly due to a reduction of their apoptotic rate. Stimulation of mitosis by ActA in both the granulosa cells which can encapsulate individual oocyte, and germ cells which still do not enter meiosis, is an alternative possibility [72]. Interestingly, ActA has been shown to promote germ cell survival and proliferation in the developing human ovary before primordial follicle formation [73]. Like in immature oocytes cocultured onto PAGCs, ActA appeared able to increase the level of GSH in the oocytes developing within the explants of the embryonic ovaries and to improve the communication between granulosa cells and oocyte by increasing the mRNA level of the *Cx37*.

The oocyte growing phase is of particular importance for subsequent embryogenesis, since at this stage gene transcription occurs. The mRNAs synthesized are required for oocyte metabolism and development or as maternal factor used in the early development of the embryo. Oocyte growth is also a critical period for establishing epigenetic marks on the female genome. In the female germ cells, many imprinted genes appear to be controlled by methylation of the DNA on their imprint control regions (ICR) [74]. In this regard, it is particularly important we find here that ActA is required to sustain *de novo* methylation occurring during oocyte growth at least for the imprinted genes analyzed (*Igf2r* and *Peg3*). At the same time, we found that *H19* gene maintained its correct unmethylated status during the culture period.

Probably the most interesting results were that about 35% of the oocytes produced in the cultures in the presence of ActA were fertilizable and about 7% of them were able to reach the morula/blastocyst stage. These last results represent a further significant step towards the development of *in vitro* system for production of mature oocytes. Further investigations on *in vitro* oogenesis and improvement of this technology will be crucial for a more comprehensive understanding of germ cell biology in general, as well as for the advancement of reproductive technologies and medicine [75].

## Supporting Information

Figure S1 **Development of immature oocytes in vitro.** (A) Representative pictures of the oocytes and PAGCs of the four experimental groups. Arrows indicate degenerating oocytes. Green cells are EGFP transgenic ones, and gray cell are normal cells. Scale bars: 100 µm. (B) Growth of the immature oocytes from 12–14-day-old mice in the four experimental groups (see, Fig. 1). Oocytes cultured onto PAGCs (DOs+PAGCs) or onto PAGCs in the presence ActA show a significant increase of diameter. (C) Intracellular GSH levels in the oocytes of the four experimental groups as above. Oocytes of the DOs+PAGCs+ActA show a significant increase of GSH levels. For comparison the GSH levels of 14 and 21 dpp oocytes are also shown. (D) Evaluation of apoptosis in the oocytes of the four experimental groups. The culture onto PAGCs results in a significant reduction of the number of apoptotic oocytes. (E) Real-time quantitative PCR analysis of *Bax* and *Bcl-2* transcripts in the oocytes of the four experimental groups. ActA causes a significant reduction of the *Bax* mRNA both in DOs and in DOs+PAGCs. (F) Real-time quantitative PCR analysis of *Cx37* transcripts in the oocytes of the four experimental groups. Oocytes cultured onto PAGCs show a significant higher level of *Cx37* mRNA; ActA causes a further increase of the transcript levels.
(TIF)

Figure S2 **Effects of ActA on PAGCs** ***in vitro*** **.** (A) Incubation for two days in the presence of ActA increases the number of BrdU positive PAGCs *in vitro*. (B) ActA decreases the number of apoptotic PAGCs cultured *in vitro* for 7 days as evaluated by the TUNEL staining.
(TIF)

Figure S3 **The histology of 10 dpp mouse ovary injected with 60 µg/kg/day ActA for 6 days.** (A, B) The quantity of the primordial follicles in 10 dpp mouse injected with 60 µg/kg/day, and the 10 and 18 dpp mice as the controls. (C) The “primitive follicle-rich region” in 10 dpp mouse injected with ActA or physiological saline for 4 days. *P<0.05; **P<0.01.
(TIF)

Figure S4 **Morphologies of oocytes and follicles within the ovarian explants in the presence of ActA.** (A, B) Primary and preantral secondary follicle-like structures around growing oocytes at different culture times (arrows). (C, D) Many oocytes grew together as ‘siamese twins’ and oocytes shared the zona pellucida (white arrows). The quantity of oocytes in the ovaries cultured *in vitro* in the presence of ActA revealed a reasonable density (E), not to be overmuch (F).
(TIF)

Figure S5 **In vitro maturation of oocytes co-cultured with PAGCs.** Examples of oocytes isolated from ovaries cultured for 28 days +ActA and transferred onto PAGCs + or – ActA in IVC at the beginning (Co-IVC 0d) and after 7 day of culture (Co-IVC 7d); oocytes onto PAGCs + or – ActA in IVC for 7 days matured in IVM at the beginning (IVM 0 h) and after 16–18 h (IVM 16–18 h).
(TIF)

Figure S6 **Characterization of the oocytes generated** ***in vitro*** **from 12.5 dpc embryonic ovaries.** (A) Percent of apoptotic oocytes (TUNEL positivity) isolated from the ovary explants after 28 days in the presence or absence of ActA. (**B,D**) Real-time quantitative PCR analysis of *Bax*, *Bcl-2* and *Cx37*. Oocytes generated in vitro from explants of ovaries of 12.5 dpc embryos after 28 days of culture in the presence or absence of ActA. (C) GSH levels in the oocytes as above.
(TIF)

Supporting Information S1 **Assay of intracellular glutathione (GSH) in oocytes.**
(DOC)

Supporting Information S2 **Proliferation and apoptosis assays of preantral granulosa cells.**
(DOC)

Supporting Information S3 **Establishment of in vitro maturation culture system for immature oocytes from prepubertal ovaries.**
(DOC)

Table S1 **Details of primers used for Real-time PCR.**
(DOCX)

Table S2 **The primers used for amplification of imprint gene by nested PCR.**
(DOCX)
