# Supplementary figures and images for: Growth of Mouse Oocytes to Maturity from Premeiotic Germ Cells In Vitro
Source: PLoS One. 2012 Jul 24;7(7):e41771. doi: 10.1371/journal.pone.0041771 (PMC3404094; doi:10.1371/journal.pone.0041771)

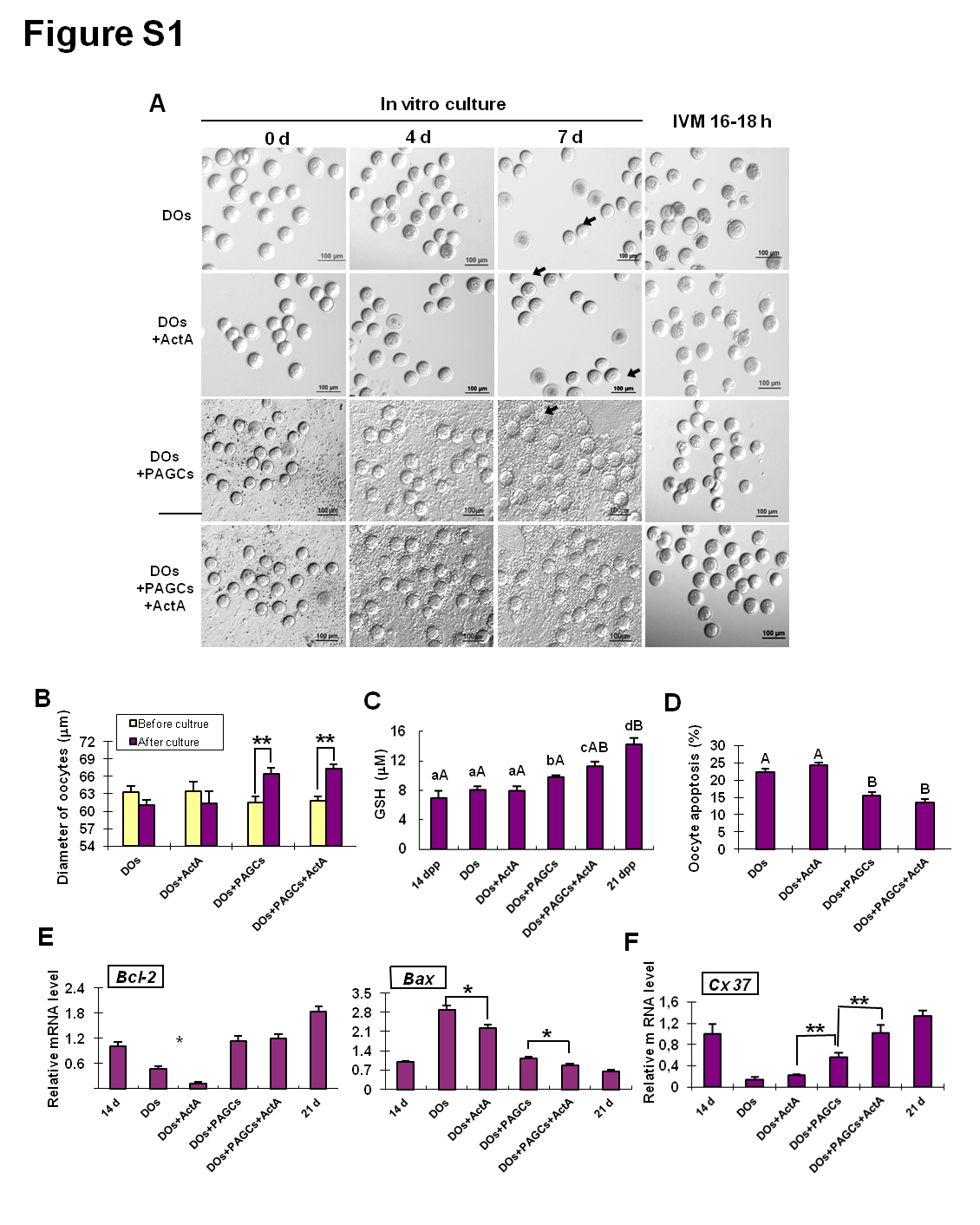

Supplement: Figure S1 — Development of immature oocytes in vitro. (A) Representative pictures of the oocytes and PAGCs of the four experimental groups. Arrows indicate degenerating oocytes. Green cells are EGFP transgenic ones, and gray cell are normal cells. Scale bars: 100 µm. (B) Growth of the immature oocytes from 12–14-day-old mice in the four experimental groups (see, Fig. 1). Oocytes cultured onto PAGCs (DOs+PAGCs) or onto PAGCs in the presence ActA show a significant increase of diameter. (C) Intracellular GSH levels in the oocytes of the four experimental groups as above. Oocytes of the DOs+PAGCs+ActA show a significant increase of GSH levels. For comparison the GSH levels of 14 and 21 dpp oocytes are also shown. (D) Evaluation of apoptosis in the oocytes of the four experimental groups. The culture onto PAGCs results in a significant reduction of the number of apoptotic oocytes. (E) Real-time quantitative PCR analysis of Bax and Bcl-2 transcripts in the oocytes of the four experimental groups. ActA causes a significant reduction of the Bax mRNA both in DOs and in DOs+PAGCs. (F) Real-time quantitative PCR analysis of Cx37 transcripts in the oocytes of the four experimental groups. Oocytes cultured onto PAGCs show a significant higher level of Cx37 mRNA; ActA causes a further increase of the transcript levels. (TIF) [file pone.0041771.s001.tif]

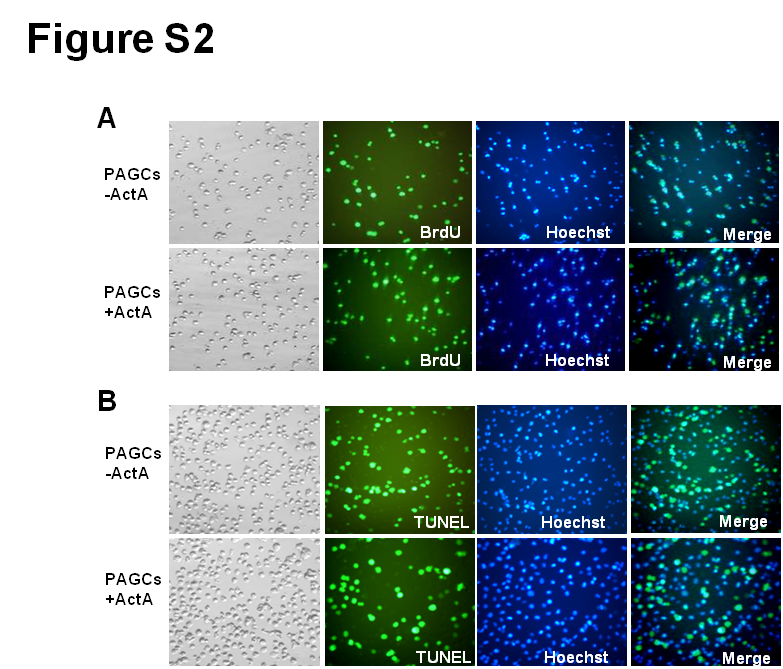

Supplement: Figure S2 — Effects of ActA on PAGCs in vitro . (A) Incubation for two days in the presence of ActA increases the number of BrdU positive PAGCs in vitro. (B) ActA decreases the number of apoptotic PAGCs cultured in vitro for 7 days as evaluated by the TUNEL staining. (TIF) [file pone.0041771.s002.tif]

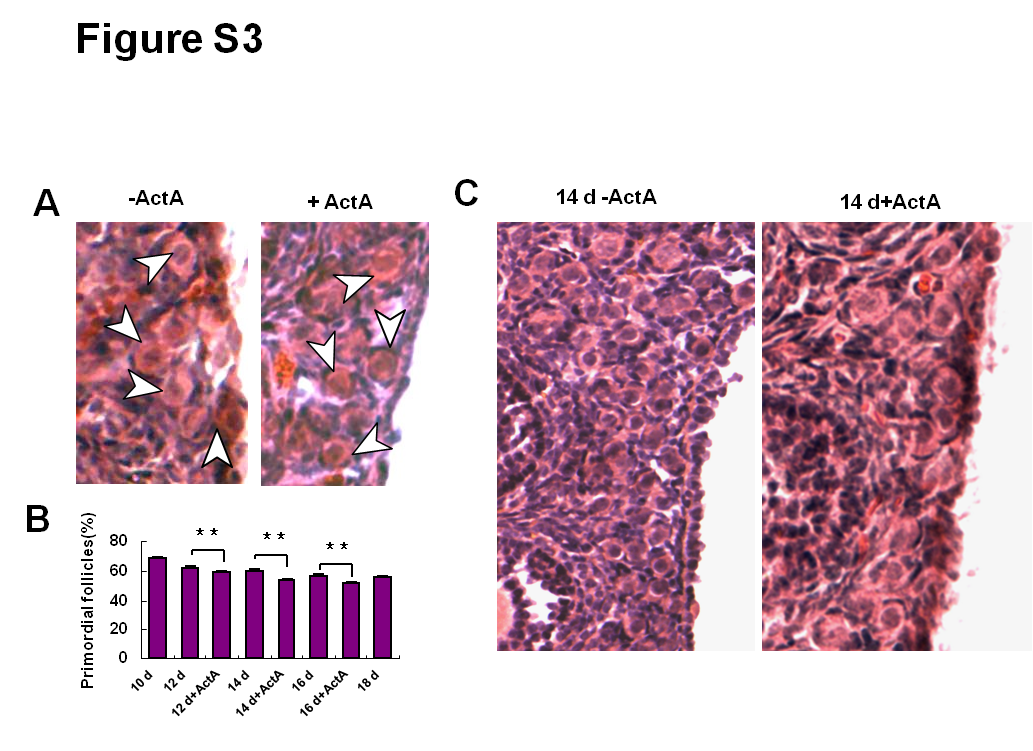

Supplement: Figure S3 — The histology of 10 dpp mouse ovary injected with 60 µg/kg/day ActA for 6 days. (A, B) The quantity of the primordial follicles in 10 dpp mouse injected with 60 µg/kg/day, and the 10 and 18 dpp mice as the controls. (C) The “primitive follicle-rich region” in 10 dpp mouse injected with ActA or physiological saline for 4 days. *P<0.05; **P<0.01. (TIF) [file pone.0041771.s003.tif]

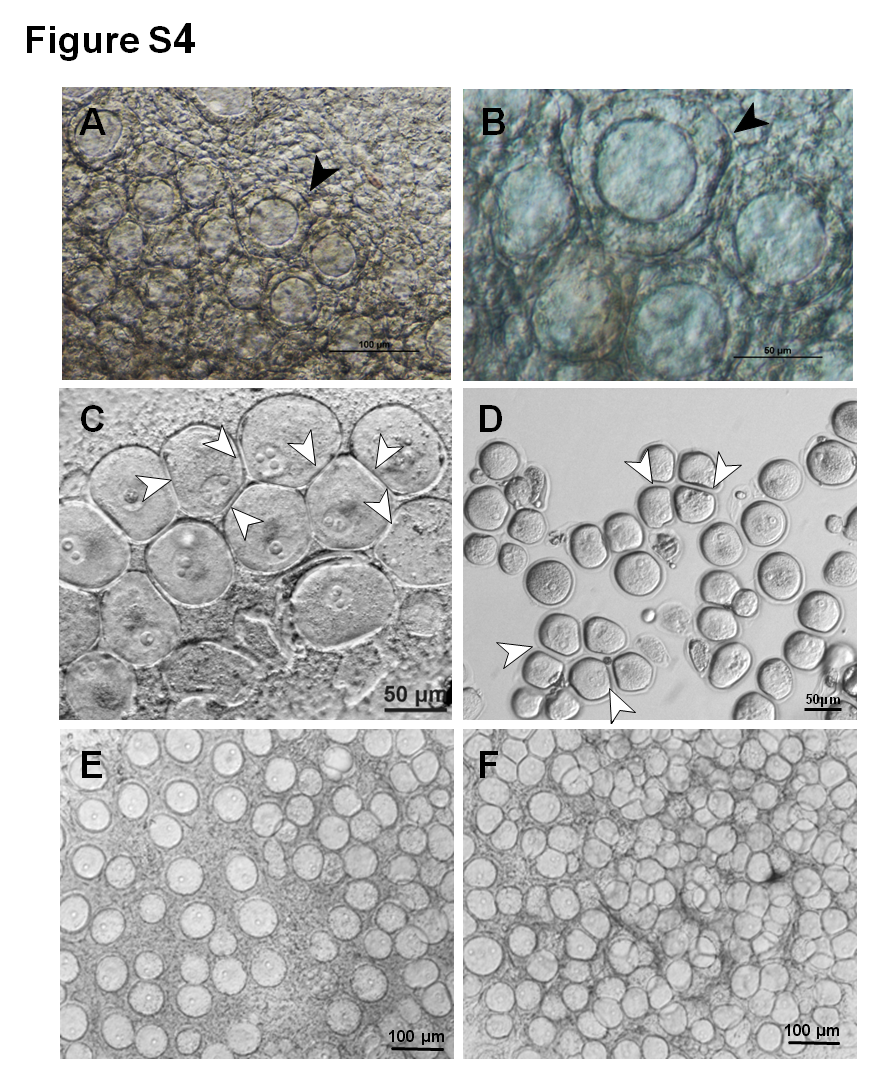

Supplement: Figure S4 — Morphologies of oocytes and follicles within the ovarian explants in the presence of ActA. (A, B) Primary and preantral secondary follicle-like structures around growing oocytes at different culture times (arrows). (C, D) Many oocytes grew together as ‘siamese twins’ and oocytes shared the zona pellucida (white arrows). The quantity of oocytes in the ovaries cultured in vitro in the presence of ActA revealed a reasonable density (E), not to be overmuch (F). (TIF) [file pone.0041771.s004.tif]

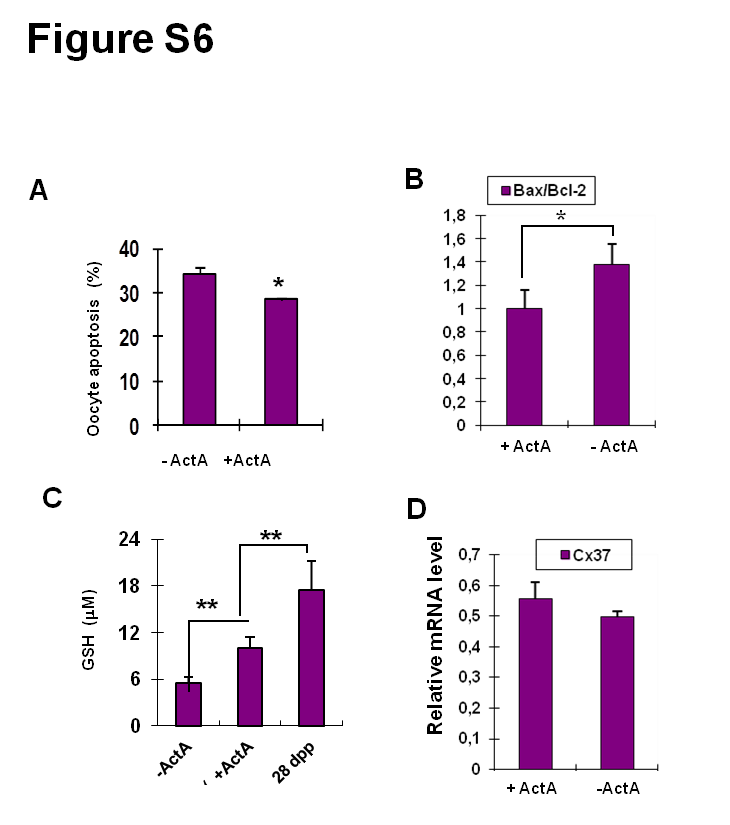

Supplement: Figure S6 — Characterization of the oocytes generated in vitro from 12.5 dpc embryonic ovaries. (A) Percent of apoptotic oocytes (TUNEL positivity) isolated from the ovary explants after 28 days in the presence or absence of ActA. (B,D) Real-time quantitative PCR analysis of Bax, Bcl-2 and Cx37. Oocytes generated in vitro from explants of ovaries of 12.5 dpc embryos after 28 days of culture in the presence or absence of ActA. (C) GSH levels in the oocytes as above. (TIF) [file pone.0041771.s006.tif]
