# Supplementary material for: Growth of Mouse Oocytes to Maturity from Premeiotic Germ Cells In Vitro
Source: PLoS One. 2012 Jul 24;7(7):e41771. doi: 10.1371/journal.pone.0041771 (PMC3404094; doi:10.1371/journal.pone.0041771)
Supplement: Supporting Information S1 — Assay of intracellular glutathione (GSH) in oocytes. (DOC) [file pone.0041771.s007.doc]

***Assay of intracellular glutathione (GSH) in oocytes***

Intracellular GSH content in oocytes was measured as described previously (1). Briefly, oocytes were collected and washed three times in FHM medium (Chemicon, MA, USA). Fifty oocytes in 5 μl distilled water were transferred into a 1.5 ml centrifuge tube, and 5 μl of 1.25 M phosphoric acid was added to the tube. Samples were frozen at -80 °C and thawed at room temperature. This procedure was repeated 5 times. The samples were stored at -80 °C until future analysis. GSH concentration in oocytes was determined by 5, 5’-dithiobis (2-nitrobenzoic acid) (DTNB)-oxidized GSH reductase recycling assay according to the manufacturer’s protocol (Beyotime). Absorbance at 405 nm was monitored by a spectrophotometer for 25 min with an interval of 5 min. The intracellular GSH concentration was defined as the ratio between the amount of GSH in each sample and the number of oocytes. All experiments were repeated 6 times and the data were expressed as mean ± SEM .

**References**

1. Dong HS, Li L, Song ZH, Tang J, Xu B, Zhai XW, Sun LL, Zhang P, Li ZB, Pan QJ, Shi QH, Shen W (2009) Premeiotic fetal murine germ cells cultured in vitro form typical oocyte-like cells but do not progress through meiosis. Theriogenology 72:219-231
