# Supplementary material for: Growth of Mouse Oocytes to Maturity from Premeiotic Germ Cells In Vitro
Source: PLoS One. 2012 Jul 24;7(7):e41771. doi: 10.1371/journal.pone.0041771 (PMC3404094; doi:10.1371/journal.pone.0041771)
Supplement: Supporting Information S2 — Proliferation and apoptosis assays of preantral granulosa cells. (DOC) [file pone.0041771.s008.doc]

***Proliferation and apoptosis assays of preantral granulosa cells***

PAGCs were collected as described above and cultured in DMEM/F12 supplemented with 10% FCS, 0.23 mM pyruvic acid, 100 U/ml penicillin G and 100 mg/ml streptomycin sulfate in a humidified atmosphere supplemented with 5% CO_2_ and air After five days of culture, PAGCs were collected and equal number of cells (10^5^ cells/well) was seeded into a 24-well plate and cultured with 30 μg/ml 5-bromo-2-deoxyuridine (BrdU) in the presence or absence of 100 ng/ml ActA for 48 hr. The BrdU incorporation assay was performed as previously described (1). Single fluorescent cells were counted on the basis of propidium iodide nuclear staining and BrdU signal. Three independent cultures were analyzed.

In order to evaluate apoptosis in PAGCs and the effect of ActA, cells were cultured for 7 days with or without ActA and subjected to the TUNEL assay according to manufacturer’s protocol (Beyotime, Haimen, China). As the negative control, cells were incubated with 50 µl of TUNEL-labeled solution without terminal transferase. The cells were photographed on a Nikon TE-2000 microscope equipped with an epifluorescent detector.

Reference

1. Dyce PW, Wen L, Li J (2006) In vitro germline potential of stem cells derived from fetal porcine skin. Nat Cell Biol 8:384-390
