# Supplementary material for: Growth of Mouse Oocytes to Maturity from Premeiotic Germ Cells In Vitro
Source: PLoS One. 2012 Jul 24;7(7):e41771. doi: 10.1371/journal.pone.0041771 (PMC3404094; doi:10.1371/journal.pone.0041771)
Supplement: Supporting Information S3 — Establishment of in vitro maturation culture system for immature oocytes from prepubertal ovaries. (DOC) [file pone.0041771.s009.doc]

**Establishment of *in vitro* maturation culture system for immature oocytes from prepubertal ovaries**

After culturing of DOs in the presence or absence of ActA, we did not observe any significant increase of diameter (61.03 ± 0.78 (n=1009), and 61.36 ± 1.24 μm (n=881), respectively). In contrast, the diameters of oocytes cocultured with PAGCs both in the presence or absence of ActA were significantly increased to a similar extent (*P* < 0.01) (66.37 ± 1.09 (n=1233), and 67.27 ± 0.78 μm (n=1120), respectively) (Fig. S1B). These diameters were, however, significantly lower than those of oocytes of comparable *in vivo* age (21-22 dpp) (n= 56; 86.47 ± 2.81 μm, *P* < 0.01).

Since increased level of GSH is considered a reliable mark of oocyte cytoplasmic maturation (1), we measured the GSH levels in the oocytes of the four groups after 6-7 days of culture. As shown in Fig. S1C, the GSH levels increased significantly only in the oocytes of DOs+ PAGCs+ ActA group (*P* < 0.01). Thus suggesting a cooperative effect of granulosa cells and ActA on the maturation of the oocyte cytoplasm.

The apoptosis of oocytes was also examined by the TUNEL method. We found that the percentage of apoptotic oocytes was significantly lower in groups with PAGCs (DOs+PAGCs, 15.50 ± 0.99% (n=235); DOs+PAGCs+ActA, 13.40 ± 1.08% (n=201)) than in groups without PAGCs (DOs, 22.30 ± 0.95% (n=291); DOs +ActA, 24.30 ± 0.68% (n=272)) (*P* < 0.01) (Fig. S1D). Meanwhile, the expression of apoptosis-related genes *Bcl-2* and *Bax* in these oocytes was evaluated by RT-PCR. As shown in Fig. S1E, while the levels of *Bcl-2* transcripts were higher in the oocytes of DOs group than that of DOs+ActA group, those of *Bax* were significantly lower in DOs+ActA and DOs+PAGC oocytes (P<0.05). In this latter, ActA appeared to reinforce the PAGC effect (*P* < 0.05).

Gap junction between the oocyte and the surrounding granulosa cells is required for proper oocyte growth and maturation (2-5). Cx37 is present in gap junctions between the oocyte and surrounding cumulus cells and has been localized at the surface of the oocytes. Thus, we examined the expression of *Cx37* genes in oocytes cultured in different groups by using real-time PCR. As shown in Supplemental Fig. 1F, mRNA expression level of *Cx37* in the DOs+PAGCs+ActA group was significantly higher than that of the DOs+PAGCs group and the groups without PAGCs (*P* < 0.01).

Taken together these results indicate that PAGCs and ActA cooperate to ensure the growth and maturation of immature oocytes *in vitro*. Moreover, they suggest that while the granulosa cells favor mainly the oocyte survival by reducing apoptosis, ActA exerts a more complex effect on the oocyte maturation, favoring both the GSH increase and the oocyte-granulosa cell communications (Fig. S1). Moreover, it appeared that most of the ActA effect on the oocytes required the presence of PAGCs, thus indicating such cells as the main target of this growth factor. However, the growth and the capability to resume meiosis (GVBD) and reach the MII stage of the immature oocytes cultured onto PAGCs plus ActA resulted still significantly smaller than the oocytes of equivalent *in vivo* chronological age (21-22 day post partum).

**References**

1. Luberda Z (2005) The role of glutathione in mammalian gametes. [Reprod Biol.](http://www.ncbi.nlm.nih.gov/pubmed?term= oocyte Luberda%2C 2005) 5(1):5-17.
2. Simon AM, Goodenough DA, Li E, Paul DL (1997) [Female infertility in mice lacking connexin 37.](http://www.ncbi.nlm.nih.gov/pubmed/9020357) Nature.385(6616):525-9.
3. Ackert CL, Gittens JE, O'Brien MJ, Eppig JJ, Kidder GM (2001) Intercellular communication via connexin43 gap junctions is required for ovarian folliculogenesis in the mouse. Dev Biol 233:258-270
4. Gittens JE, Mhawi AA, Lidington D, Ouellette Y, Kidder GM (2003) [Functional analysis of gap junctions in ovarian granulosa cells: distinct role for connexin43 in early stages of folliculogenesis.](http://www.ncbi.nlm.nih.gov/pubmed/12620892) Am J Physiol Cell Physiol 284(4):C880-887.
5. Veitch GI, Gittens JE, Shao Q, Laird DW, Kidder GM (2004) [Selective assembly of connexin37 into heterocellular gap junctions at the oocyte/granulosa cell interface.](http://www.ncbi.nlm.nih.gov/pubmed/15138288) J Cell Sci 117(Pt 13):2699-2707.
