# Supplementary material for: Growth of Mouse Oocytes to Maturity from Premeiotic Germ Cells In Vitro
Source: PLoS One. 2012 Jul 24;7(7):e41771. doi: 10.1371/journal.pone.0041771 (PMC3404094; doi:10.1371/journal.pone.0041771)
Supplement: Table S1 — Details of primers used for Real-time PCR. (DOCX) [file pone.0041771.s010.docx]

**Supplemental Table 1** Details of primers used for Real-time PCR

| **Gene** | **Primers** | **Annealing temperature** (℃) | **Fragment size (bp)** |
| --- | --- | --- | --- |
| ***Bcl-2*** | F: 5’-ACC ACC TAG AGC CTT GGA TCC -3’  R: 5’-TCT CGG CTG CTG CAT TGT T -3’ | 60 | 186 |
| ***Bax*** | F: 5’ -ATGCGTCCACCAAGAAGCTGAG-3’  R: 5’ -CCCCAGTTGAAGTTGCCATCAG-3’ | 60 | 162 |
| ***Cx37*** | F: 5’-AAG AGCGGT TGC GGC AGA AAG AGG G-3’  R: 5’-GCA GGT TGA GCA CCA GGG AGA TGA C-3’ | 65 | 324 |
| ***actin*** | F: 5’-TCG TGG GCC GCT CTA GGC AC-3’  R: 5’-TGG CCT TAG GGT TCA GGG GG-3’ | 60 | 243 |
