# Supplementary material for: Growth of Mouse Oocytes to Maturity from Premeiotic Germ Cells In Vitro
Source: PLoS One. 2012 Jul 24;7(7):e41771. doi: 10.1371/journal.pone.0041771 (PMC3404094; doi:10.1371/journal.pone.0041771)
Supplement: Table S2 — The primers used for amplification of imprint gene by nested PCR. (DOCX) [file pone.0041771.s011.docx]

**Supplemental Table 2** The primers used for amplification of imprint gene by nested PCR

| **Gene** | **PCR** | **Primers** | **Fragment size (bp)** |
| --- | --- | --- | --- |
| *Igf2r*  *Peg3*  *H19* | 1st  2nd  1st  2nd  1st  2nd | F: 5＇-TAGAGGATTTTAGTATAATTTTAA-3＇  R: 5＇-CACTTTTAAACTTACCTCTCTTAC-3＇  F: 5＇-GAGGTTAAGGGTGAAAAGTTGTAT-3＇  R: 5＇-CACTTTTAAACTTACCTCTCTTAC-3＇  F: 5＇-TTGTTGATGTTAATTTTGTGTTTTGGTG-3＇  R: 5＇-TCAACCTTATCAATTACCCTTAAAAACC-3＇  F: 5＇-TTTTGTAGAGGATTTTGATAAGGAGGTG-3＇  R:5＇-CCCCAAACACCATCTAAACTCTACAAAC-3＇  F: 5'-GAGTATTTAGGAGGTATAAGAATT-3'  R: 5'-ATCAAAAACTAACATAAACCCCT-3'  F: 5'-GTAAGGAGATTATGTTTATTTTTGG-3'  R: 5'-CCTCATAAAACCCATAACTAT-3' | 490  288  423 |
